# Dairy products viscosity estimated by laser speckle correlation

**DOI:** 10.1371/journal.pone.0203141

**Published:** 2018-09-07

**Authors:** Dmitry D. Postnov, Flemming Moller, Olga Sosnovtseva

**Affiliations:** 1 Department of Biomedical Sciences, Copenhagen University, Copenhagen, Denmark; 2 Department of Biomedical Engineering, Boston University, Boston, United States of America; 3 Physical Food Science, DuPont Nutrition Biosciences Aps, Edwin Rahrs Vej 38, DK-8220 Brabrand, Denmark; University of Montreal, CANADA

## Abstract

Dairy products exhibit several physical properties that are crucial to define whether we like the food or not: firmness, creaminess, thickness, or lightness. Viscosity changes the flow properties of food and influences the appearance and the consistency of a product; this control variable is important in most production stages—manufacture, processing, and storage. Viscosity of heterogeneous products at a given temperature depends on its composition and physical state of its substances. Although rheology provides a method to access the product viscosity, it lacks non-contact full-field monitoring. We apply a simple correlation analysis of laser speckle images to evaluate viscosity properties of dairy products. Our approach ensures robust measurements with high degree of detectability.

## Introduction

We often accept food products on the basis of their texture: How spreadable and creamy a food product is. This affects taste perception [1]. Moreover, products with different viscosity but with similar palatability, macronutrient composition and energy density significantly differs in their intake: a case study in obesity [2]. Main factors—used ingredients, quality of raw material, technological process—can influence rhelogical properties of dairy products. The fat concentration, physico-chemical properties of fat globules and protein content affect viscosity. These compounds can be modified during thermal and mechanical operations that can significantly change rheological characteristics. For these reasons, food rheology is an important quality control tool during and after food manufacture and processing.

The market requires to design new food and beverages that are accepted by consumers. Food industry studies and predicts consumers’ perceptions of food through instrumental measurements [3–6]. There are different rheological tests in the food industry [7, 8]. By applying a controlled force (or strain) and measuring the resulting force (or strain), it is possible to access rheological properties of a material. The rheometer is a research-grade analytical instrument that measures viscous, elastic, and viscoelastic properties of liquids, semisolids, and solids. Capillary viscometers, falling-ball viscometers, rotational and oscillatory rheometers, among others, are used to perform rheological measurements [9]. Dairy products are normally physical homogeneous, but physical or chemical changes (e.g. sedimentation, creaming, wheying off) can occur during storage and limit the shelf life. Some defects can be seen and detected with imaging techniques but viscosity changes due to age gelation or fat crystallization cannot be visualized with traditional visualization techniques.

Over the last decades, development of fast digital acquisition and data processing technologies advanced laser speckle techniques for medical applications in the retina [10], skin [11], mesenterium [12], the kidney [13], and the brain [14]. When coherent light scatters from a medium, it produces a random interference pattern termed speckle pattern. The intensity of speckle patterns fluctuates due to movement of scattering particles in the medium. This leads to a blurring of the speckle pattern, to a reduction in the local speckle contrast and, thus, to a coding velocity distribution.

Recently, Hajjarian and Nadkarni [15, 16] nailed a new approach—laser speckle rheology—to assess tissue viscoelasticity by measuring the time constant of fluctuations of laser speckle intensity. Speckle fluctuations are highly sensitive to Brownian movement of scattering particles; they are influenced by the viscoelasticity of the medium and are analyzed by means of the speckle intensity autocorrelation curves.

We applied a simple correlation analysis of laser speckle images to evaluate viscosity properties of dairy products without direct contact and modifications of the medium. The advantages of the suggested approach over traditional methods include minimal sample preparation, contact-free assessment, fast acquisition time, and simultaneous characterization of rheological properties as well as physical defects.

## Materials and methods

### Imaging

Dairy product samples were provided by DuPont Nutrition Biosciences Aps, Denmark. The samples were produced by a combination of 38% fat cream, skimmed milk and viscosity increasing agent CREMODAN^®^ 719 at different concentrations. Cremodan^®^ 719 is a blend of food-grade stabilisers of hydrocolloids (E412, E415, E407) in the powder form. The hydrocolloids increases viscosity with increasing concentrations. In the used levels they should also prevent protein aggregation and resulting sedimentation. The final set consisted of 27 samples with 0.2, 1.95, 3.7% of fat and 0.000, 0.015, 0.03, 0.045, 0.06, 0.075, 0.09, 0.105, 0.12% of CREMODAN^®^ 719.

The laser module LDM785 controlled by the diode driver Thorlabs CLD1011LP delivered coherent infrared light to the sample; the laser power density was about 20 mW/cm^2^ [17]. Back-scattered light was collected by CMOS camera Basler acA2000-165umNIR that operated at a rate of 400 (low frequency LF mode) or 2000 (high frequency HF mode) frames per second. In the LF mode, an image of 400 × 200 pixels was recorded with 2 ms of exposure time; in the HF mode, an image of 400 × 25 pixels was recorded with 0.5 ms of exposure time. To deliver light to the camera, VZM^™^ 1000i lens (Edmund Optics) were used at 10x magnification. The field of view and focal distance remained the same during experiments. Temperature of the samples and in the room was kept constant at about 22 °C.

Three imaging sessions were performed in LF mode and one session in HF mode. The samples were selected randomly. Each recording lasted for one minute; the first 10 seconds were removed from the analysis as a transient period.

The samples were analysed using a Physica MCR 301 rheometer (Anton Paar, Austria) and a DG26.7 double gab geometry. A standard viscosity profile was made starting at a shear rate of 0.1 s^−1^ and increasing to 200 s^−1^ using a logarithmic ramp. 25 points were recorded using a fixed measuring duration of 20 s pr. point. The samples were stored at refrigerated temperature (app. 5 °C) and tempered at 10 °C in the measuring system for 5 minutes before measurements. The analyses were performed at 20 °C. The measured data were fitted to the linear model and shear viscosity *μ* was extracted assuming that samples are Newtonian fluid at low fat and CREMODAN^®^ 719 concentrations. R-squared was used as a statistical measure of how close the data are to the fitted regression line.

### Data analysis

**Fig 1:**
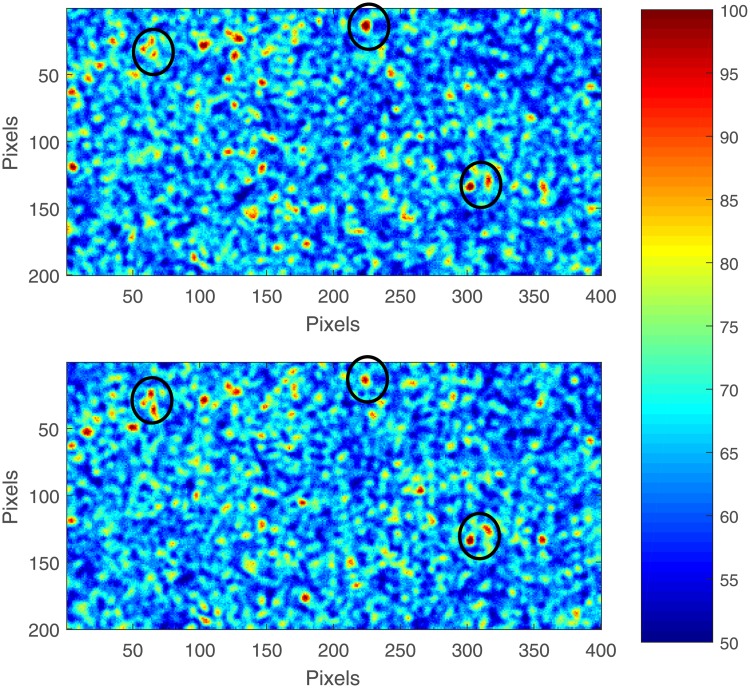
Speckle images. Two consecutive frames for a sample with 3.7% of fat and 0.12% of CREMODAN^®^ 719. Color codes intensity of scattered light. Black circles mark exemplary regions where the speckle pattern remains unchanged and, thus, increases correlation.

Scattering particles—fat globules or large proteins—create a speckle pattern that changes due to Brownian motion (Fig 1). Speed of particles and, thus, change of the speckle pattern are mainly defined by temperature, particle size, and medium viscosity. At constant temperature, particle size remained the same in the samples with the same fat concentration; in the samples with different CREMODAN^®^ 719 concentrations particle speed was defined only by medium viscosity. To estimate viscosity variations by means of laser speckle imaging, we suggested two approaches.

#### Correlation time

The first approach is based on the analysis of intensity autocorrelation function for speckle dynamics [14, 18]:

$$g_{mn}^{\left(2\right)}\left(\tau \right)=\frac{\left\langle I_{mn}\left(t\right)I_{mn}\left(t+\tau \right)\right\rangle }{{\left\langle I_{mn}\left(t\right)\right\rangle }^{2}},$$ (1)

where *m*, *n* are pixel indexes, *I* is speckle intensity, *t* is time corresponding to the first frame, and *τ* is a time lag. *g*^(2)^ was calculated for samples over 100 time points and 50 lags. In the absence of static scattering *g*^(2)^ is related to the field temporal autocorrelation function *g*^(1)^ [14] as

$$g^{\left(2\right)}\left(\tau \right)=1+\beta {\left|g^{\left(1\right)}\left(\tau \right)\right|}^{2},$$ (2)

where *β* accounts for loss of correlation related to the speckle size and light source properties.

$$g^{\left(1\right)}\left(\tau \right)=exp\left(-{\left(\frac{\tau }{\tau_{c}}\right)}^{n}\right),$$ (3)

where *n* takes values of 0.5, 1 or 2 depending on the scattering regime and the motion properties [19]. Correlation time *τ*_*c*_ is a key parameter in laser speckle theory; it is inversely proportional to the speed of scattering particles [14, 20]. We estimate correlation time by substituting *τ* with *τ*_*c*_ and 0 in Eqs 2 and 3 and numerically solving the following equation with respect to *τ*_*c*_:

$${\left\langle g^{\left(2\right)}\left(\tau_{c}\right)\right\rangle }_{mn}=\frac{{\left\langle g^{\left(2\right)}\left(0\right)\right\rangle }_{mn}-1}{e^{2}}+1,$$ (4)

where *e* is mathematical constant approximately equal to 2.718. As correlation time inversely proportional to the particle speed, we expect that it will increase with larger viscosity which is known to decrease particles speed in Brownian motion.

#### Frames correlation

The second approach is based on 2-dimensional linear correlation between frames:

$$C\left(t,\tau \right)=\frac{\sum_{m}\sum_{n}\left(I_{mn}\left(t\right)-\bar{I}\left(t\right)\right)\left(I_{mn}\left(t+\tau \right)-\bar{I}\left(t+\tau \right)\right)}{\sqrt{\left(\sum_{m}\sum_{n}{\left(I_{mn}\left(t\right)-\bar{I}\left(t\right)\right)}^{2}\right)\left(\sum_{m}\sum_{n}{\left(I_{mn}\left(t+\tau \right)-\bar{I}\left(t+\tau \right)\right)}^{2}\right)}},$$ (5)

where $\bar{I}\left(t\right),\bar{I}\left(t+\tau \right)$ are mean intensities of corresponding frames. Similar to the Pearson correlation coefficient, *C*(*t*, *τ*) shows degree of linear correlation and is equal to 1 if *I*(*t*) and *I*(*t* + *τ*) are linearly related and to 0 if there is no correlation. Since motion of particles causes random changes in speckle pattern, correlation reflects relative number of particles moved between two frames. This metric depends on the time between frames *τ*. If it is too long, the frames become independent of each other; if it is too short, there might be not enough motion to change the speckle pattern. For recordings in LF mode, we used only consecutive frames, thus, *τ* was fixed at the value of 2.5 ms. For recordings in HF mode the time lag *τ* was varied from 0.5 ms to 25 ms to obtain linear correlation curves similar to *g*^(2)^. The linear correlation was calculated for all possible pairs of frames in each recording. Values above and below one standard deviation from the median of the linear correlation were rejected to avoid imaging artifacts such as skipped frames. Remaining values were averaged to provide a reliable estimation of linear correlation. Resulting average takes values from 0 to 1 (negative linear correlation is unlikely for random changes of the speckle pattern): 0 corresponds to high particles mobility (low viscosity) and 1 to low particles mobility (high viscosity). We call it *viscosity index*:

$$V_{\tau }=\bar{C_{\tau }}\left(\right|C_{\tau }\left(t\right)-M_{\tau }\left|<\right|M_{\tau }-\sigma_{\tau }\left|\right),$$ (6)

where *M*_*τ*_ and *σ*_*τ*_ are median value and standard deviation for all correlation coefficients calculated at the selected *τ*. *C*_*τ*_(|*C*_*τ*_(*i*) − *M*_*τ*_| < |*M*_*τ*_ − *σ*_*τ*_|) denotes all correlation coefficients *C*_*τ*_ at *τ* that satisfy this filtering conditions. Viscosity indexes calculated from data at LF mode were analyzed for significant difference between samples with different CREMODAN^®^ 719 concentrations. Unlike correlation time (Eq 4), the linear frame correlation (Eq 5) does not directly depend on particle speed, but shows stability degree of speckle pattern that is linked to particle mobility.

#### Robustness analysis

We used Student’s test for independent samples to detect a minimal difference in CREMODAN^®^ 719 concentrations with 95% confidence. To estimate robustness of the identified changes we calculated detectability index [17]:

$$D=|\bar{v}\left(c_{1}\right)-\bar{v}\left(c_{2}\right)|/(0.5\times (\sigma_{v}\left(c_{1}\right)+\sigma_{v}\left(c_{2}\right))),$$ (7)

where $\bar{v}$ and *σ*_*v*_ are mean and standard deviation of an analyzed variable. *c*_1_ or *c*_2_ are CREMODAN^®^ 719 concentrations to be compared. Analyzed variable can be either *τ*_*c*_ (Eq 4) or *V* (Eq 6).

## Results

**Fig 2:**
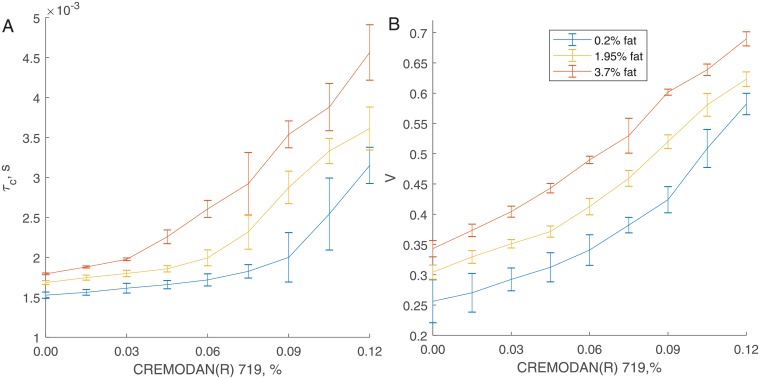
Characterization of samples. Correlation time *τ*_*c*_ (A) and viscosity index *V* (B) calculated for all samples in LF mode. Both characteristics increase with increasing CREMODAN^®^ 719. This is related to the rise of viscosity.

Fig 2 depicts correlation time and viscosity indexes calculated for the data recorded in LF mode. CREMODAN^®^ 719 increases viscosity of dairy products: the larger concentration of CREMODAN^®^ 719, the stronger correlation between the frames, longer correlation time (A), and higher viscosity index (B). Variations of the correlation time within the same sample increase with increasing CREMODAN^®^ 719 concentration while curves are well separated for different fat concentrations. Variations of the viscosity index are low and decrease with increasing fat concentration. Increasing fat concentration results in increased number of large, slow moving particles; this makes viscosity estimation more precise and robust.

We introduced detectability index (Eq 7) to evaluate how reliable detected differences are. Table 1 compares detectability index for *τ*_*c*_ (Eq 4) or *V* (Eq 6) for all CREMODAN^®^ 719 concentrations. One can see that *D*_*v*_ tends to increase with increasing both fat and CREMODAN^®^ 719 concentrations; such increase is observed in 81% and 71% of compared pairs, respectively. $D_{\tau_{c}}$ demonstrates similar behavior with increasing fat concentration (75% of compared pairs) but does not show steady improvement with increase of CREMODAN^®^ 719 concentration (48% of compared pairs). This indicates that autocorrelation function *g*_(2)_ (Eq 1) is more vulnerable to acquisition noise.

**Table 1:** Detectability index. It estimates reliable differences that the proposed approaches can distinguish.

CREMODAN^®^ 719, %	fat 0.2%		fat 1.95%		fat 3.17%	
	*D*_*v*_	$D_{\tau_{c}}$	*D*_*v*_	$D_{\tau_{c}}$	*D*_*v*_	$D_{\tau_{c}}$
0.000-0.015	0.42	0.94	2.25	2.24	2.54	7.30
0.015-0.030	0.87	1.09	2.49	1.47	3.19	6.53
0.030-0.045	0.93	0.78	2.51	2.46	4.58	5.48
0.045-0.060	1.16	0.93	3.61	1.98	6.68	3.63
0.060-0.075	2.17	1.33	3.50	2.05	2.29	1.26
0.075-0.090	2.43	0.89	4.98	2.65	4.25	2.21
0.090-0.105	3.20	1.43	4.04	2.51	5.08	1.47
0.105-0.120	2.99	1.80	2.75	1.32	4.82	2.13

**Fig 3:**
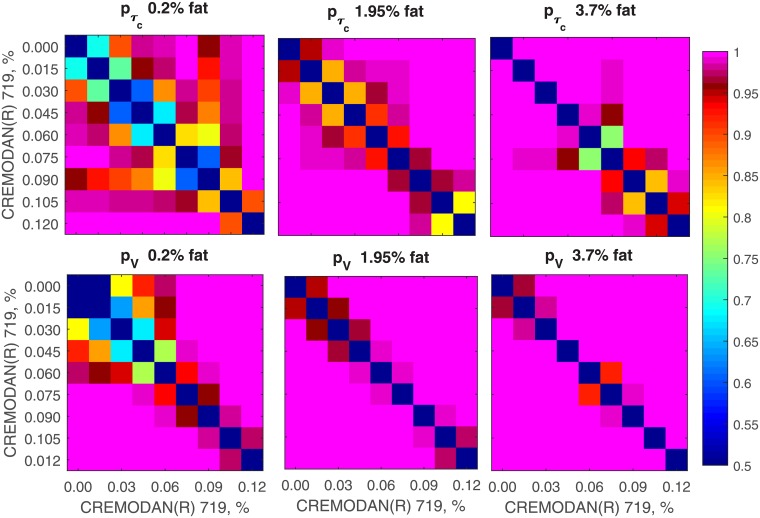
Robustness of the method. Color codes confidence with which different CREMODAN^®^ 719 concentrations can be distinguished in the samples with the same fat concentration. Confidence was calculated on the basis of Student’s t-test for *τ*_*c*_ (top panel) and *V* (bottom panel). Dark red and magenta colors correspond to the confidence above 95%. Viscosity index allows for 95% confidence detection in a wider range of concentrations.

To estimate robustness and precision of the method, we calculated confidence at which one can distinguish different CREMODAN^®^ 719 concentrations by means of correlation time and viscosity index (Fig 3). In most cases variations are low enough to assess 0.015% changes in CREMODAN^®^ 719 concentration with 95% confidence. The most difficult case is to make precise estimations at low concentration of fat and CREMODAN^®^ 719 (left panel). This limitation is related to the fast movements and low number of large particles.

**Fig 4:**
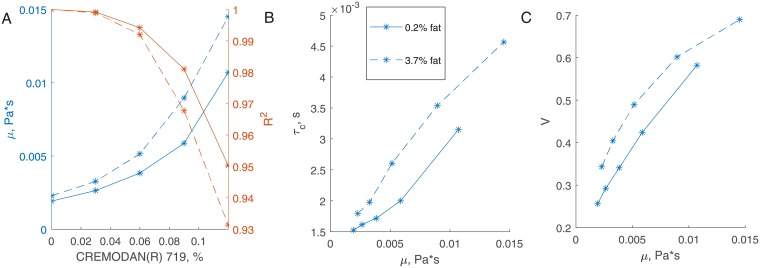
Viscosity estimation. (A) Shear viscosity *μ* obtained by a linear fit of rheometery measurements (left axis) and *R*^2^ goodness of fit (right axis). With increasing CREMODAN^®^ 719 and fat concentrations the fit quality decreases because of non-Newtonian properties of the diary product. *τ*_*c*_ (B) and *V* (C) as functions of *μ* show almost linear correlation—particularly for low-fat samples. Solid lines correspond to 0.2% fat concentration, dashed lines to 3.7%.

To validate the method we compared viscosity estimated from laser speckle measurements with shear viscosity. Shear viscosity *μ* was obtained by linear fitting of shear stress (measured by rheometer) to shear rate (Fig 4A). Shear viscosity was measured for samples with 0.2 and 3.7% fat concentrations and 0.000, 0.03, 0.06, 0.09, 0.12% CREMODAN^®^ 719 concentrations. Pearson correlation coefficient between *μ* and *τ*_*c*_ was 0.9856 and 0.9947 at 0.2% and 3.7% fat concentrations, respectively; for viscosity index *V* they were 0.9975 and 0.9716. This points to almost linear relation between laser speckle and traditional rheology measurements (Fig 4B and 4C). Non-linear dependency between shear viscosity and laser speckle measurements is expected at higher values of fat or CREMODAN^®^ 719 concentrations as non-Newtonian properties of the dairy product are more pronounced (Fig 4A).

**Fig 5:**
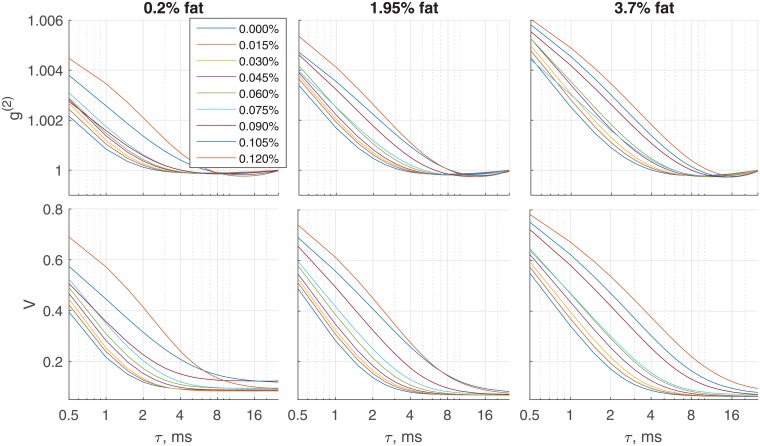
Correlation curves. Autocorrelation *g*^(2)^ (top panel) and viscosity index *V* (bottom panel) versus time lag. Time lag is defined as a delay between frames. Color codes CREMODAN^®^ 719 concentrations. Although curves have slightly different forms, they reach minimum within 5–10 ms.

Correlation time *τ*_*c*_ is one of the parameters that define the shape of autocorrelation curve *g*^(2)^. Correlaion time corresponds to a specific time lag ((Eq 4)) while viscosity index *V* depends on time lag (sampling frequency). To show how *V* changes for different sampling frequencies and how well *τ*_*c*_ describes the shape of *g*^(2)^, we used the data obtained in HF mode. Fig 5 represents corresponding correlation curves. Both *g*^(2)^ and *V* reach their minimum around a time lag *τ* of 5–10 ms. This implies that a sampling frequency below 200 frames per second can not capture observed variations of viscosity; the consecutive frames are not correlated. A better separation of samples is possible if the correlation is close to one; this will require much higher frame rate (about 20000 frames per second), higher laser power and low signal-to-noise ratio. Another observation is that the form of curves slightly varies for different CREMODAN^®^ 719 concentrations (e.g, some curves cross each other). In such cases *τ*_*c*_ or *V* are not sufficient to estimate viscosity. But this effect is mainly determined by the presence of noise; longer recordings or appropriate method to compensate the acquisition noise could solve the problem.

## Conclusion

We showed that variations of dairy product viscosity can be estimated by simple laser speckle analysis—without special high frequency cameras and frequency response analysis. To estimate viscosity we suggested two approaches:

- Estimation of correlation time *τ*_*c*_ (Eq 4) from speckle autocorrelation function (Eq 1) gives linear relation to viscosity for Newtonian liquids;
- Estimation of viscosity index *V* (Eq 6) from frame-to-frame linear correlation (Eq 5) evaluates a degree of stability of speckle patterns that is related to particle mobility.

Correlation analysis of laser speckle data is sufficient for robust measurements of viscosity variations; it detects 0.015% changes in CREMODAN^®^ 719 concentration with 95% confidence in most cases (44% for 0.2% fat concentration, 88% for 1.95% and 3.7% fat concentrations). Precision of the method rises with increasing fat concentration of the product.

We should mention several benefits of frame-to-frame linear correlation versus speckle autocorrelation: (i) computational implementation is simpler and less time consuming, (ii) its value does not dependent on the speckle contrast, and (iii) it is less vulnerable to acquisition noise and temporal artifacts. This results in better separation of viscosity variations. Simple modification of experimental protocol—increasing length of recording or imaging speed—can significantly improve detectability of viscosity changes.

Robustness of the results and reliable separation of samples with respect to CREMODAN^®^ 719 and fat concentrations may allow for mapping viscosity index to the absolute values and for applying the method to a wide range of products. Simple speckle correlation analysis opens perspectives to monitor structural and physical changes of food products during manufacture and storage.
